# The Applications of Plant Polyphenols: Implications for the Development and Biotechnological Utilization of *Ilex* Species

**DOI:** 10.3390/plants13233271

**Published:** 2024-11-21

**Authors:** Gong Cheng, Yuxiao Yan, Bingsong Zheng, Daoliang Yan

**Affiliations:** 1 State Key Laboratory of Subtropical Silviculture, Zhejiang A&F University, Hangzhou 311300, China; cgg@stu.zafu.edu.cn (G.C.); bszheng@zafu.edu.cn (B.Z.); 2 College of Life Science, Anqing Normal University, Anqing 246133, China; yanyuxiao0115@sina.com

**Keywords:** *Ilex*, plant polyphenols, development and utilization, application prospects

## Abstract

Plants belonging to the *Ilex* species are distinguished by their rich composition of diverse phenolic compounds and various bioactive substances, which demonstrate dual functionalities in therapeutic applications and health promotion. In recent years, these plants have garnered significant interest among researchers. While the application scope of plant polyphenols (PPs) is extensive, the exploration and utilization of holly polyphenols (HPs) remain comparatively underexplored. This article reviews the research advancements regarding the predominant phenolic compounds present in commonly studied *Ilex* species over the past five years and summarizes the application studies of PPs across various domains, including pharmacological applications, food technology, health supplements, and cosmetic formulations. The objective of this review is to provide insights into the systematic research and development of HPs, offering references and recommendations to enhance their value.

## 1. Introduction

Plants belonging to the *Ilex* species within the Aquifoliaceae family are predominantly found in temperate and subtropical regions, with an estimated total of approximately 810 species worldwide [1]. In China, more than 200 species have been identified, with the majority distributed across the Yangtze River Basin and southern regions [2]. Notable *Ilex* species, such as *Ilex latifolia* Thunb., *Ilex cornuta* Lindl. and Paxton, *Ilex pubescens* Hook. and Arn., and *Ilex kudingcha* C.J. Tseng, are recognized for their considerable medicinal value. These plants exhibit a range of beneficial properties, including antiviral effects, lipid-lowering capabilities, anti-cancer activity, cardiovascular protection, and modulation of gut and immune functions, which have contributed to their long-standing use in traditional Chinese medicine [3,4,5,6,7,8,9,10,11].

In addition, in South American countries such as Argentina, Brazil, and Paraguay, Yerba Mate, derived from the leaves of *Ilex paraguariensis* A.St.-Hil., has gained popularity as a healthy alternative to coffee and tea. Its long-term consumption has been associated with various health benefits, including anti-obesity and anti-diabetic effects, as well as cardiovascular protection [12,13,14,15,16,17]. Similarly, beverages made from *Ilex guayusa* Loes. and *Ilex vomitoria* Aiton are favored in the Amazon and the United States for their medicinal and health-promoting properties [18,19,20].

Beyond their medicinal applications, *Ilex* species significantly contribute to landscape architecture, providing benefits such as dust suppression, noise reduction, and the absorption of toxic gases [21,22,23]. Additionally, *Ilex verticillata* (L.) A. Gray and *Ilex aquifolium* L. are commonly utilized for Christmas decorations in various countries, particularly in Europe, due to their striking red fruits [24].

PPs are a class of common secondary metabolites with a polyphenolic structure, predominantly found in various plant organs, including leaves, roots, fruits, stems, and bark. These compounds play a significant role in the growth, development, and defense of plants [25,26]. The structure of PPs is intricate and diverse, with three primary subclasses based on their chemical composition: flavonoids, phenolic acids, and other non-flavonoid compounds [27]. The presence of phenolic compounds was first identified in tea leaves, and to date, over 8000 phenolic compounds and their derivatives have been isolated and characterized from natural sources [28,29].

PPs are abundant resources that are derived from environmentally sustainable sources and are relatively easy to extract. These compounds exhibit a range of bioactive functions, including antioxidant, anti-inflammatory, and antibacterial activities, as well as the potential for disease prevention and treatment. In recent years, PPs have gained significant attention within the medical, food, and industrial sectors, attracting interest from both researchers and consumers. While *Ilex* species are known to contain polyphenolic compounds, their potential for value utilization remains underexplored, with most of the existing literature focusing primarily on their applications in medicine and food. Therefore, this paper aims to review recent advancements in the research and application of PPs across various fields to identify promising developmental pathways for HPs. This review seeks to provide references for future development and systematic research, while also offering new insights for enhancing the utilization of *Ilex* species resources.

## 2. Polyphenolic Compounds in Common *Ilex* Species

Most *Ilex* species contain a diverse array of polyphenols. The concentration of these HPs can vary significantly due to several factors, including environmental conditions, extraction methodologies, and the growth status of the plants. In China, the polyphenol content in *I. kudingcha* is generally found to be higher than that in *I. latifolia* across nearly all production areas [30]. The primary polyphenolic compounds identified in *Ilex* species include chlorogenic acid and its derivatives, along with other minor compounds such as neochlorogenic acid, rutin, and quercetin. These polyphenolic compounds are recognized as key pharmacologically active molecules that impart anti-inflammatory, antibacterial, and detoxifying properties to various plants (Table 1).

## 3. Application of PPs

### 3.1. Antibacterial and Anti-Inflammatory Effects

Inflammation is a defensive response initiated by the body in reaction to adverse stimuli, including mechanical injury and microbial infection. However, prolonged inflammatory responses can lead to various pathological conditions. PPs have been identified as safe and eco-friendly natural anti-inflammatory agents. For instance, polyphenols derived from Tibetan tea effectively inhibit the elevation of inflammatory factor levels and ameliorate the inflammatory response in mice subjected to a high-fat, high-sugar diet [45]. Additionally, green tea polyphenols exhibit strong inhibitory effects on the production of pro-inflammatory cytokines and reactive oxygen species (ROS), as well as on the induction of the TLR4 signaling pathway, thereby demonstrating significant anti-inflammatory properties [46,47].

In *I. paraguariensis*, polyphenol extracts have been shown to enhance the expression levels of antioxidant-related genes and increase the activity of superoxide dismutase (SOD), effectively mitigating oxidative damage in retinal pigment epithelium (RPE) cells. This mechanism not only provides therapeutic and preventive benefits against age-related macular degeneration (AMD) and other retinal degeneration-related diseases but also modulates the expression of genes associated with inflammatory immune cells, cell motility, and intercellular interactions. Consequently, this contributes to the treatment and prevention of inflammatory and cardiometabolic diseases [48,49]. Furthermore, the chlorogenic acid and quercetin present in these extracts exhibit distinct neuroprotective effects in both the forebrain and midbrain, demonstrating promising preventive capabilities against neurotoxic and inflammatory damage linked to lung cancer [50]. Polyphenols extracted from *I. rotunda* and *I. asprella* have been shown to effectively inhibit the production of nitric oxide (NO) in RAW264.7 cells, demonstrating potent anti-inflammatory activity [51,52]. Polyphenols derived from *I. latifolia* exert their effects by inhibiting the expression levels of mitogen-activated protein kinases (MAPKs), specifically extracellular signal-regulated kinase (ERK) and c-Jun N-terminal kinase (JNK), as well as nuclear factor kappa B (NF-κB). This inhibition leads to a reduction in the production of NO and pro-inflammatory cytokines. Furthermore, these polyphenols play a regulatory role in the antioxidant enzyme system and possess potent antioxidant properties, which may help prevent diseases associated with oxidative stress and inflammation [32,53].

Moreover, PPs are recognized as natural antimicrobial agents with extensive applications in inhibiting microbial growth. Phenolic compounds extracted from *Ruta graveolens* L. significantly inhibit protein denaturation and the growth of Staphylococcus aureus, showcasing considerable anti-inflammatory and antibacterial activities [54]. Future research on the anti-inflammatory activity of HPs should reference the applications of other plants, with the aim of further elucidating the mechanisms underlying anti-inflammatory and antibacterial effects, particularly the specific molecular signaling pathways involved. Additionally, exploring the potential development of HPs into anti-inflammatory and antibacterial drugs, functional foods, or dietary supplements will be of significant interest.

### 3.2. Anti-Aging and Anti-Fatigue Effects

Polyphenolic compounds present in highland barley tea have been demonstrated to alleviate palmitic acid-induced mitochondrial damage in skeletal muscle in vitro, thereby mitigating the effects of skeletal muscle aging [55]. Additionally, polyphenols derived from *A. grossedentata* enhance the antioxidant capacity within the liver of mice, improve bone metabolism during the aging process, and regulate gut microbiota, collectively contributing to the attenuation of aging [56]. Furthermore, research indicates the potential of *I. kudingcha* polyphenol extracts in aging prevention, as these extracts significantly alleviate organ aging induced by D-galactose in mice. This is achieved through mechanisms such as improving the morphology of aging liver, skin, and spleen tissues, reducing the production of inflammatory cells and upregulating the expression of antioxidant-related genes and proteins [57].

Disruption of the body’s energy coordination can result in a heightened sense of fatigue, with prolonged fatigue potentially precipitating related diseases. PPs exhibit notable anti-fatigue properties, for instance, polyphenols extracted from *Eleutherococcus sessiliflorus* (Rupr. and Maxim.) S.Y. Hu significantly prolong swimming endurance in mice by increasing levels of liver glycogen, muscle glycogen, and lactate dehydrogenase activity, while simultaneously decreasing concentrations of urea nitrogen, malondialdehyde (MDA), and lactate [58].

The robust antioxidant capacity of PPs mitigates oxidative stress-induced cellular damage, thereby delaying the onset of cellular aging. The anti-fatigue effects are primarily mediated through enhanced energy metabolism, promotion of the clearance and recovery of fatigue-related metabolites, and enhancement of immune function. Future research should further elucidate the specific mechanisms through which HPs exert their anti-aging and anti-fatigue effects while also exploring their applications in functional foods, dietary supplements, cosmetics, and sports nutrition products, thus contributing significantly to human health and quality of life.

### 3.3. Anti-Cancer Effects

Cancer represents a major threat to human health, with the quest for effective control and prevention methods being a shared aspiration among patients and medical professionals alike. PPs have emerged as potent anti-cancer agents, exhibiting a range of effects through diverse mechanisms, including the inhibition of cancer cell proliferation, suppression of metastasis, and induction of apoptosis in malignant cells. For instance, green tea polyphenols have demonstrated significant inhibitory effects on the proliferation of breast cancer MDA-MB-231 cells, underscoring their potential as anti-cancer agents [46]. Additionally, dietary sources of PPs, such as spinosin-Na—a polyphenolic compound extracted from Ziziphi Spinosae Semen—have shown considerable promise in inhibiting the proliferation of colorectal cancer cells, suggesting its potential development as a dietary preventive measure against colorectal cancer [59]. Currently, *I. latifolia* polyphenols have been shown to suppress the proliferation of human lung cancer cells (A549) and promote apoptosis through the modulation of the PI3K-Akt signaling pathway [7].

Advancements in scientific technologies are poised to enhance future research, allowing the application of sophisticated methodologies such as molecular biology, genomics, and proteomics. These approaches will facilitate a deeper understanding of the interactions between HPs and cancer cells, ultimately providing substantial support for the development of more effective and safer anti-cancer therapeutics. Given the successful cases of PPs in the prevention and treatment of cancer, future research and applications of HPs in cancer therapy are expected to lead to significant breakthroughs.

### 3.4. Regulation of Intestinal Health and Lipid-Lowering Effects

The gut microbiota plays a vital role in digestion, metabolism, and vitamin synthesis within the human body. PPs can modulate the gut microenvironment by regulating intestinal digestive enzyme activity, promoting lipid and glucose metabolism, and enhancing energy expenditure. These mechanisms collectively contribute to lipid-lowering and anti-obesity effects, as well as reductions in blood glucose levels. For instance, green tea polyphenols have been demonstrated to significantly downregulate the expression of genes related to lipogenesis while upregulating genes and enzymatic activities associated with lipid transport and catabolism, thereby alleviating obesity and decreasing blood lipid levels [60,61]. Additionally, polyphenols derived from the leaves of *Morus alba* L. effectively inhibit α-glucosidase activity and enhance the expression of genes implicated in lipid metabolism, thus promoting glucose absorption and metabolism to exert hypoglycemic effects [62]. Furthermore, polyphenolic compounds extracted from the leaves of *Cycas circinalis* L. significantly lower blood glucose levels in diabetic mice and improve their lipid profiles, indicating their potential for medicinal and nutritional food development [63].

Enhancing the abundance of beneficial gut microbiota and maintaining microbial diversity are effective strategies for promoting gut health and preventing intestinal diseases. The polyphenols found in Fuzhuan brick tea regulate the gut microbiome by decreasing the Firmicutes-to-Bacteroidetes ratio and increasing the abundance of Bifidobacteriaceae, thereby significantly mitigating the onset of obesity [64]. Similarly, polyphenols from red-osier dogwood have been recognized for their ability to promote gut health and are frequently utilized as prebiotics in the animal and food industries [65]. Moreover, polyphenolic compounds present in the leaves of *Ceratonia siliqua* L. can diminish the expression of pro-inflammatory cytokines in the colon, adipose tissue, and spleen of mice, while also reducing NO production [66]. Additionally, polyphenols from *A. argyi* have been shown to enhance the levels of beneficial bacteria and favorable metabolites, demonstrating promising therapeutic effects against obesity and colonic inflammation induced by dextran sulfate sodium (DSS) [67].

In *I. kudingcha*, polyphenol extracts have been shown to enhance the activities of endogenous enzymes, including SOD and glutathione peroxidase (GSH-PX), in rats. These extracts inhibit the production of lipid peroxidation products, such as MDA, as well as serum lipopolysaccharide (LPS) levels and metabolic enzyme activity. Consequently, this leads to a reduction in blood lipid levels and decreased absorption of fats and carbohydrates. Additionally, these extracts may reduce the abundance of Erysipelothrix species, enhance the diversity of the gut microbiota, and demonstrate lipid-lowering and weight-reducing effects [64,68]. Given the increasing prevalence of obesity, hyperglycemia, hyperlipidemia, and gut-related diseases attributed to irregular lifestyles and dietary habits, the application of HPs in regulating gut health, lowering lipid levels, reducing blood glucose, and preventing obesity offers considerable promise, particularly as public awareness of health continues to grow.

### 3.5. Beauty and Whitening Effects

Long-term exposure to ultraviolet (UV) radiation is associated with substantial damage to human skin, manifesting as sunburn, hyperpigmentation, moisture depletion, and a reduction in skin elasticity and firmness. Recent studies have demonstrated that polyphenolic extracts from *Moringa oleifera* Lam. can effectively modulate oxidative stress in photo-aged skin, thereby enhancing tissue morphology and collagen structure. This modulation confers protective effects against photo-aging induced by UV radiation [69]. Similarly, *I. kudingcha* polyphenol extracts play a critical role in mitigating UV-induced skin damage by lowering serum MDA activity, reducing levels of inflammatory factors (IL-6, IL-1β, and TNF-α), and decreasing hydrogen peroxide levels in skin tissue, thereby protecting damaged skin cells [70].

Tyrosinase, an enzyme that catalyzes the oxidation of tyrosine to produce melanin, plays a critical role in various biochemical pathways, and dysregulation of this enzyme can result in skin disorders. Notably, polyphenols derived from *Rubus chingii* Hu have exhibited significant inhibitory effects on both intracellular and extracellular tyrosinase activity, leading to a marked reduction in intracellular melanin synthesis [71]. Furthermore, traditional whitening cosmetics often pose adverse effects on both health and the environment due to their production processes and raw materials. In contrast, safe and non-toxic natural cosmetics formulated from PPs have garnered increasing attention in research, both domestically and internationally. For example, hand creams enriched with polyphenols from Pu’er tea are characterized by their non-irritating properties, distinctive tea fragrance, appealing coloration, and superior moisturizing capabilities [72].

The unique chemical structure and biological activities of PPs underscore their considerable potential for application in beauty and whitening cosmetics. These compounds exhibit a range of beneficial properties, including antioxidant, anti-inflammatory, antibacterial, whitening, and anti-aging effects, which collectively enhance skin quality and mitigate damage from external stressors such as UV radiation. The cosmetics industry is witnessing a discernible shift toward green and sustainable practices, with natural and eco-friendly ingredients, particularly PPs, being increasingly incorporated into various beauty and whitening products, including creams, serums, and masks. Anticipated trends suggest that consumer acceptance and market demand for cosmetics containing PPs will continue to grow. Future research should aim to elucidate the mechanisms underlying the antioxidant, anti-inflammatory, and whitening effects of HPs, facilitating the development of more targeted and effective beauty products that align with consumer preferences for natural, safe, and efficacious skincare solutions.

### 3.6. Animal Husbandry and Breeding Field

The antioxidant and antibacterial properties, along with various other biological activities of PPs, have increasingly positioned them as a focal point of research within the livestock industry. These compounds are commonly utilized as feed additives, contributing to enhanced animal growth and improved production performance through their anti-inflammatory and intestinal protective functions. For instance, the incorporation of 80 g·d^−1^ of bamboo leaf polyphenol extract into the diets of beef cattle has been shown to optimize gut microbiota composition and alter ruminal fermentation characteristics. This modification subsequently enhances the metabolic utilization of feed and the digestibility of fiber components, leading to increased daily weight gain in beef cattle [73]. Moreover, the inclusion of *Eucommia ulmoides* Oliv. leaf polyphenol extract in pig diets significantly increases muscle fiber density while concurrently reducing muscle fiber diameter, thereby improving muscle fiber composition and enhancing meat quality [74].

In dairy cattle, the addition of green tea polyphenols during the early lactation period has been demonstrated to positively influence lipid metabolism and mitigate reductions in feed intake due to summer heat stress, ultimately resulting in increased milk production. When included in dog food, these polyphenols effectively inhibit fatty acid synthesis and transport, thereby modulating lipid metabolism and exerting a lipid-lowering effect that aids in controlling body weight, which is particularly beneficial for the treatment or prevention of obesity in pet dogs [75,76,77]. In the context of aquaculture, the addition of 1% *M. oleifera* leaf polyphenol extract to the feed of *Oreochromis niloticus* significantly enhances the activity of antioxidant enzymes in the liver and kidneys while upregulating the expression levels of anti-inflammatory genes. This intervention effectively ameliorates liver and kidney damage induced by abamectin [78]. Furthermore, the incorporation of 500 mg/kg of *I. chinensis* leaf polyphenol extract into broiler diets has been found to enhance intestinal digestion and absorption, improve gut health, increase serum SOD activity, and decrease serum MDA concentrations. These effects collectively contribute to improved antioxidant capacity and growth performance in broilers [79]. Moreover, incorporating polyphenols from *I. latifolia* into the feed of piglets with intestinal injury significantly enhances the antioxidant capacity of the intestinal mucosa, improves the histological and functional characteristics of the intestinal lining, and alleviates damage to intestinal epithelial cells caused by ferroptosis by regulating the expression of cell-related genes and proteins [80].

In conclusion, PPs have significant potential in the livestock and aquaculture industries, as they can reduce feed costs while effectively improving animal performance and product quality. However, appropriate inclusion levels should be carefully selected when used as feed additives. Future applications of HPs in the livestock industry could draw insights from the applications of other plant species, including the relevant fields and animal species involved. Additionally, further experimental studies are required to provide a stronger scientific foundation for the practical application of HPs in production.

### 3.7. Food Industry

In comparison to traditional chemically synthesized preservatives, PPs are increasingly utilized in food preservation due to their environmentally friendly nature, safety, and abundant availability, along with their high antibacterial and antioxidant efficacy. However, it is crucial to recognize that the effectiveness of preservation and consumption is influenced by the concentration of added PPs. For instance, in a 20 °C environment, a concentration of 0.4 g·L^−1^ of *Lycium chinense* Mill. leaf polyphenol extract exhibited superior preservation efficacy for *Lycopersicon esculentum* Mill. compared to concentrations of 0.2 and 0.6 g·L^−1^ [81]. Moreover, the judicious addition of *Elaeagnus rhamnoides* (L.) A. Nelson leaf polyphenols to apple juice effectively mitigates browning, minimizes vitamin C degradation, and achieves preservation while maintaining flavor and nutritional value. However, excessive polyphenol incorporation may detrimentally impact the quality, taste, and color of the juice [82].

The integration of PPs during food processing can enhance the quality, flavor, texture, and shelf life of food products. Specifically, the incorporation of 0.03% *Zanthoxylum bungeanum* Maxim. leaf polyphenol extract in the processing of salted silver carp effectively reduces lipid oxidation levels while imparting distinctive flavors, colors, and textures [83]. Additionally, the inclusion of *Solenostemma argel* (Delile) Hayne leaf polyphenols in chicken meatballs can decrease mold counts, improve lipid oxidation status, and enhance antioxidant activity without adversely affecting sensory characteristics, thereby effectively prolonging shelf life [84]. In sausage production, the addition of PPs can improve color quality, slow down oxidation rates, enhance microbial quality, and modify the microbial community structure, ultimately extending the shelf life of the product [85,86].

In the beverage industry, adding 0.1% total polyphenol extract from dandelion tea to plant-based beverage formulations has yielded positive feedback regarding taste, color, clarity, and aroma, imparting a unique flavor to the drink [87]. By incorporating lotus leaf polyphenols into traditional yellow wine brewing methods, a special yellow wine can be produced that exhibits higher antioxidant capacity compared to conventional yellow wine [88]. PPs also show significant potential in seafood preservation; for instance, adding polyphenols from *Platostoma palustre* (Blume) A.J. Paton and *Vaccinium* sp. leaves during the storage of shrimp and squid balls can effectively inhibit lipid oxidation and microbial growth, slow down protein degradation, and reduce total bacterial counts, thereby delaying spoilage and extending shelf life [89,90].

Recent studies have demonstrated that PPs can act as natural plant-based antioxidants in edible oils, significantly enhancing the oxidative stability of fats. These compounds offer several advantages, including high safety, non-toxicity, and potent antioxidant capabilities. For instance, the incorporation of 0.07% *Thymus vulgaris* L. leaf polyphenol extract into soybean oil markedly improves its oxidative stability [91]. Similarly, the addition of 0.01% to 0.02% *Psidium guajava* L. leaf polyphenols into lard and peanut oils effectively reduces the oxidation rate of these fats [92]. In conclusion, PPs have found extensive applications within the food industry, functioning not only as natural antioxidants to prolong the shelf life of food products but also in enhancing their taste, color, and nutritional value. Furthermore, there exists significant potential for the development of functional foods and dietary supplements tailored to address specific health needs in the future.

Food packaging made from PPs demonstrates notable antibacterial and antioxidant properties, effectively inhibiting the proliferation of microorganisms and preventing the oxidation of nutrients within food products. The incorporation of polyphenol extracts derived from *M. alba* leaves into pectin-based edible films can significantly improve their mechanical properties, as well as their antioxidant and antibacterial activities. Furthermore, the natural preservatives, antioxidants, and antibacterial agents obtained from this extract enhance the quality of fresh-cut fruits, exhibiting substantial effects in delaying fruit softening and preserving color integrity [93,94]. Additionally, applications of HPs in the food industry have been documented. For instance, the incorporation of polyphenol extracts from *I. paraguariensis* into the fabrication process of corn starch films significantly enhances the antioxidant properties, heat resistance, and mechanical strength of the films, rendering them suitable as environmentally friendly packaging materials [95].

## 4. Conclusions and Prospects

**Figure 1:**
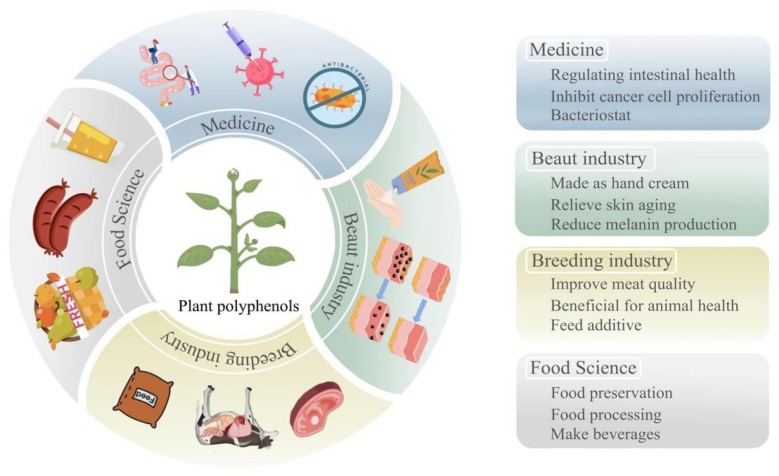
Current status of PP development and utilization. The blue section indicates applications in the field of medicine. The green section indicates applications in the field of beauty industry. The yellow section indicates applications in the field of breeding industry. The gray section indicates applications in the field of food science.

PPs are abundant in nature, readily accessible, cost-effective, and renewable, showcasing a wide range of biological activities that underscore their substantial potential and value for various applications. Presently, PPs are employed across multiple sectors, including medicine, food, industry, and healthcare (Figure 1).

Recent advancements in the separation and purification techniques for HPs have been achieved through various efficient extraction methods, including solvent extraction, microwave-assisted extraction, and ultrasonic-assisted extraction. The favorable bioactivity of HPs indicates substantial application potential across multiple fields, such as food, pharmaceuticals, and landscaping. Notably, in the medical field, its diverse biological activities, including antioxidant, anti-inflammatory, antitumor, and antibacterial effects, hold promise for the prevention and treatment of a wide range of diseases. Nonetheless, significant challenges persist that hinder the advancement of HPs, particularly concerning the enhancement of their development, increased recognition, and broader application. This paper delineates the obstacles faced in the development and utilization of HPs, while exploring several avenues that may facilitate their future advancement and application, informed by the current landscape of PP research and development.

First, concerning raw materials, *Ilex* species are widely distributed worldwide and encompass numerous species. However, current research on the extraction, characterization, and subsequent studies of HPs is predominantly limited to a select few species, including *I. paraguariensis*, *I. latifolia*, and *I. kudingcha*. Future investigations should broaden their focus to encompass a wider array of *Ilex* species. Such expansion not only has the potential to enhance the sources of HPs but also facilitates the discovery of novel polyphenolic compounds, thereby establishing a robust foundation for future development and utilization.

**Figure 2:**
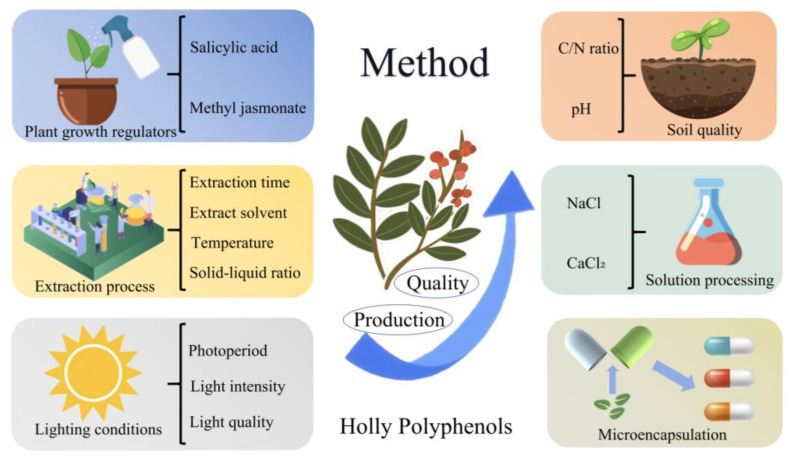
Methods to enhance the quality, yield, and utilization rate of HPs. The blue section indicates the use of plant growth regulators. The yellow section represents improvements in extraction techniques. The gray section denotes alterations in light conditions. The orange section reflects enhancements in soil quality. The green section signifies the application of solution treatments. The brownish-yellow section indicates the utilization of microencapsulation technology.

Second, with regard to quality, yield, and utilization rates, there remains considerable potential for improvement in both the quality and yield of HPs. Strategies to enhance the quality and yield of HPs may include the application of plant growth regulators [96], regulation of light conditions [26], optimization of extraction processes [97,98,99], solution treatments [100], and improvements in soil quality [101] (Figure 2). Furthermore, studies have demonstrated that encapsulating PPs within microcapsules can effectively protect the active ingredients from degradation due to external conditions, thus facilitating improved storage and transportation. This approach enhances the antioxidant properties and controlled release of PPs. For instance, polyphenol nanoparticles derived from *Eucalyptus robusta* Sm. leaves exhibited superior solubility and release rates in simulated in vitro digestion experiments when compared to free *E. robusta* leaf polyphenols, significantly improving the stability and bioavailability of the polyphenols [102]. The microencapsulation of PPs offers high stability and utilization rates, positioning it as a focal point for future research.

Thirdly, with regard to bioactivity and future development trends, current research on the bioactivity and functional characteristics of HPs remains limited. Consequently, *Ilex* species have predominantly been explored in medicine and food science. Future research should prioritize a deeper investigation into the composition and structural properties of HPs, with the aim of uncovering additional bioactive components and elucidating the underlying mechanisms of action. This will not only broaden the scope of HPs’ potential applications but also enhance their feasibility for development in diverse fields.

Fourthly, in the context of resource conservation and sustainable development, *Ilex* species represent a valuable source of medicinal plant resources, highlighting the importance of their conservation and sustainable management. It is essential to intensify efforts in monitoring and assessing these resources, alongside developing well-structured harvesting and cultivation strategies to ensure their long-term sustainability. Furthermore, research on HPs should encourage interdisciplinary collaboration, facilitating the continued advancement of both fundamental and applied research for HPs’ development.

## Figures and Tables

**Table 1:** The main polyphenols and their biological activity in common *Ilex* species.

Source	The Main Polyphenols	Biological Activity	Literature
*I. latifolia*	1-Caffeoylquinic acid,	Antioxidant activity	[31,32]
	dicaffeoylquinic acids		
*I. cornuta*, *I. kudingcha*	Chlorogenic acids,	Preventing gastric injury, antioxidant activity, antibacterial activities	[33,34,35]
	neochlorogenic acid,		
	cryptochlorogenic acid,		
	isochlorogenic acid A, B, C		
*I. paraguariensis*	3-caffeoylquinic acid,	Antibacterial and antiviral activities, cardiovascular protective effect, antioxidant activity, anti-diabetes, anti-obesity	[36,37,38,39]
	5-caffeoylquinic acid,		
	tannins,		
	rutin,		
	4-caffeoylquinic acid,		
	3,5-dicaffeoylquinic acid		
*I. vomitoria*	1-Caffeoylquinic acid,	Anti-inflammatory activity	[40,41]
	dicaffeoylquinic acids,		
	rutin		
*I. guayusa*	Hydroxycinnamic acid,	Antioxidant activity, anti-inflammatory activity	[42,43,44]
	quercetin-3-O-hexose,		
	chlorogenic acids		

## Data Availability

No new data were created or analyzed in this study. Data sharing is not applicable to this article.
